# Mitonuclear Coevolution in Bumblebees (*Bombus*): Genomic Signatures and Its Role in Climatic Niche Adaptation

**DOI:** 10.1093/gbe/evaf123

**Published:** 2025-06-13

**Authors:** Leonardo Tresoldi Gonçalves, Pedro Henrique Pezzi, Maríndia Deprá, Elaine Françoso

**Affiliations:** Programa de Pós-Graduação em Genética e Biologia Molecular, Departamento de Genética, Universidade Federal do Rio Grande do Sul, Porto Alegre, RS, Brazil; Programa de Pós-Graduação em Genética e Biologia Molecular, Departamento de Genética, Universidade Federal do Rio Grande do Sul, Porto Alegre, RS, Brazil; Programa de Pós-Graduação em Genética e Biologia Molecular, Departamento de Genética, Universidade Federal do Rio Grande do Sul, Porto Alegre, RS, Brazil; Centre for Ecology, Evolution and Behaviour, Department of Biological Sciences, School of Life Sciences and the Environment, Royal Holloway University of London, Egham TW20 0EX, UK

**Keywords:** Hymenoptera, local adaptation, mitochondrial DNA, mitonuclear interactions, nuclear compensation, thermogenesis

## Abstract

Mitochondria play a central role in cellular respiration, but require close coevolution with the nuclear genome for proper function. This process, termed mitonuclear coevolution, is poorly understood on species-level evolutionary timescales, despite its role in speciation. Here, we investigate mitonuclear coevolution in bumblebees (*Bombus*), a group of ecologically diverse pollinators with rapid mitochondrial (mt) DNA evolution. Leveraging genomic data from a comprehensive set of 55 bumblebee species, we quantified the evolutionary rate correlation (ERC) between mt genes and nuclear genes that interact with mitochondria (N-mt). We found a strong ERC between mt and N-mt genes, but not among mt genes and random nuclear genes, supporting the mitonuclear coevolution hypothesis. Additionally, we found the strength of mitonuclear ERC seems to be consistent across bumblebee lineages, contrasting with observations in other taxa. Finally, bumblebee species from colder environments showed increased mt evolutionary rates relative to both N-mt genes and random nuclear genes. This suggests potential implications to bumblebee climatic niche adaptation and the thermoregulation of cold-adapted species, possibly driven by selection for enhanced mt function to sustain thermogenesis and flight in low-temperature environments. Our findings are discussed considering the dynamics of mitonuclear coevolution in bumblebees and its potential role in shaping their adaptation to diverse ecological niches.

Significance Cellular respiration relies on a coordinated interaction between mitochondrial (mt) and nuclear genes involved in mt function (N-mt), yet the evolutionary dynamics of these interactions remain poorly understood. Our study in bumblebees, a diverse and ecologically important group, provides strong evidence supporting mitonuclear coevolution, as we found a significant correlation between mt and N-mt gene evolutionary rates. We also show that environmental factors, particularly cold climates, influence mtDNA evolution, suggesting selection for enhanced mt function to support thermogenesis and flight at low temperatures. These findings help bridge a critical gap in understanding how mitonuclear interactions shape evolutionary adaptation and could have implications for predicting how species respond to environmental change.

## Introduction

**Fig. 1.**
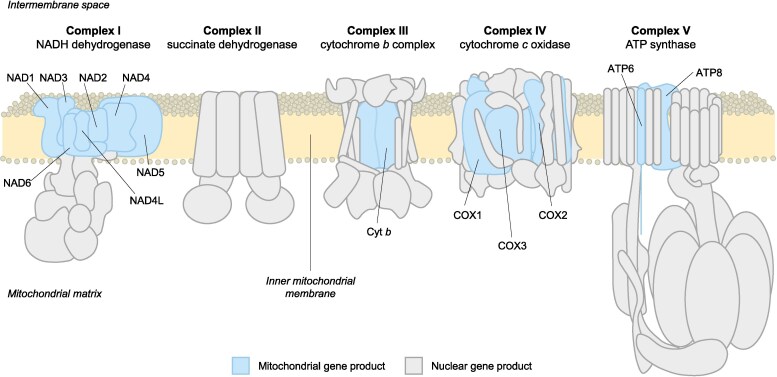
Subunit composition of the mitochondrial electron transport chain in animals, highlighting the interplay between nuclear-encoded (gray) and mitochondrial-encoded (blue) protein subunits. These complexes are embedded in the inner mitochondrial membrane (yellow). Mitochondrial-encoded proteins are core components of Complexes I, III, IV, and V, where they co-function with nuclear-encoded subunits to drive oxidative phosphorylation. Notably, Complex II consists solely of nuclear-encoded subunits. Adapted from Hahn and Zuryn (2019).

Mitochondria play a central role in providing energy to maintain cell function in eukaryotes. These organelles have their own genome, encoding proteins essential for the electron transport system, which produces ATP via oxidative phosphorylation (OXPHOS). OXPHOS involves four protein complexes (Complexes I to IV or CI to IV) that generate a proton gradient across the inner mitochondrial (mt) membrane, along with an ATP synthase (Complex V or CV) that utilizes this gradient to synthesize ATP. However, most of OXPHOS proteins are encoded in the nuclear genome, resulting in OXPHOS complexes that are a chimera of both mt and nuclear-encoded (N-mt) subunits (with the exception of CII, which is strictly nuclear-encoded) (Fig. 1). Therefore, coordination between the products of both genomes is vital for effective energy production. Incompatibilities between mt and N-mt genes can be lethal or reduce fitness (Dowling et al. 2007; Barreto and Burton 2013; Camus et al. 2017; Barreto et al. 2018; Biot-Pelletier et al. 2023; Bettinazzi et al. 2024), influencing the evolution and ecology of eukaryotes (Hill 2015; Hill et al. 2019).

Distinct dynamics characterize these two genomes in bilaterian animals: mtDNA lacks recombination, has uniparental inheritance, has a smaller effective population size (*N_e_*), and shows higher mutation rates (Ballard and Whitlock 2004). Nevertheless, both nuclear and mt genomes must evolve in concert to maintain OXPHOS, a process termed mitonuclear coevolution (Rand et al. 2004). A testable prediction derived from this hypothesis is the mitonuclear evolutionary rate correlation (ERC), where evolutionary rates align between mt and N-mt genes (de Juan et al. 2013). Previous research across animal lineages, including holometabolous insects, demonstrated a robust ERC between mt and N-mt genes, particularly for proteins that directly contact (Yan et al. 2019). However, most studies have focused on deeply divergent taxa (Weaver et al. 2022), leaving it unclear whether ERC persists at shorter evolutionary timescales, especially if speciation events are accompanied by episodes of adaptive mitonuclear coevolution.

Mitonuclear coevolution has been largely understudied in natural populations despite its important role in various evolutionary processes, from disease resistance to emergence of new species (Holmbeck et al. 2015; Bernardo et al. 2019; Rank et al. 2020). In ectothermic animals like insects, variation in environmental temperature influences the thermodynamics of OXPHOS biochemical reactions (Simčič et al. 2014), while oxygen availability directly affects the efficiency in ATP synthesis and free radical production (Fuhrmann and Brüne 2017). Therefore, important environmental factors such as temperature and oxygen availability modulate mtDNA evolution and are usually the determining factor in the success of mitonuclear coevolution (Hill 2015). This underscores how the interaction between these genomes exerts a strong selective pressure, favoring combinations that maintain OXPHOS function and local adaptation (Burton 2022).

One key hypothesis explaining how mitonuclear coevolution is maintained is nuclear compensation. This hypothesis posits that because mtDNA accumulates mutations at a faster rate than nuclear DNA, selection favors compensatory changes in N-mt products to preserve mt function. As a result, N-mt genes are expected to exhibit an elevated ratio of nonsynonymous to synonymous substitutions (*d_N_*/*d_S_*), frequently interpreted as evidence of positive selection (Shen et al. 2010; Garvin et al. 2011; Sahm et al. 2017; Barreto et al. 2018) or relaxed purifying selection (Zwonitzer et al. 2023). Support for nuclear compensation has been documented across diverse taxa, including arthropods (Barreto and Burton 2012; Meiklejohn et al. 2013; Barreto et al. 2018), mammals (Osada and Akashi 2012), and yeast (Lee et al. 2008), where N-mt genes often show signatures of adaptive substitutions (but see, e.g. Piccinini et al. 2021; Weaver et al. 2022). Furthermore, this effect is predicted to be stronger in N-mt genes coding for proteins that directly interact with mt-encoded proteins—for instance, those that make physical contact within the structure of OXPHOS complexes (Fig. 1)—as these sites are under greater selective pressure to maintain protein–protein interactions (Yan et al. 2019).

In this study, we focus on bumblebees (*Bombus*), a genus comprising approximately 270 species critical for pollination in both natural and agricultural ecosystems (http://www.nhm.ac.uk/bombus). Despite their conserved morphology, bumblebees thrive across diverse thermal environments, from lowland tropics to alpine tundra, suggesting substantial variation in mt function and evolution. Previous work has shown that bumblebee species show a wide range in climate-related traits like thermal tolerance, elevational distribution, and metabolic rates, often reflecting local adaptation to environmental niches (Martinet et al. 2015; Oyen et al. 2016; Jackson et al. 2020; Gonzalez et al. 2022). Recent genomic and transcriptomic studies have also uncovered signatures of selection associated with altitude, temperature, and oxygen availability in several *Bombus* lineages (Jackson et al. 2018, 2020; Liu, Jin et al. 2020; Liu, Zhao et al. 2020; Heraghty et al. 2022; Liang et al. 2022; Eldem et al. 2025). Similar to other Hymenoptera, bumblebees exhibit an accelerated mtDNA evolution (Lin et al. 2019; Gonçalves et al. 2024). Lineages with rapidly evolving mtDNA should in theory exhibit similar rapid evolution in N-mt genes, increasing the likelihood of compensatory evolution and mitonuclear mismatch (Havird and Sloan 2016). Despite this, mtDNA is still often overlooked in studies of bumblebee adaptation, which tend to focus primarily on the nuclear genome. Given their elevated mt substitution rates and their ability to generate heat for flight in cold environments—a process demanding intense ATP mobilization (Masson et al. 2017)—bumblebees represent an excellent model for investigating mitonuclear interactions in the context of climatic niche adaptation.

Using genomic data from a diverse set of bumblebee species, we built a robust species tree using ultraconserved element (UCE) loci, which served as a framework to investigate patterns of mitonuclear coevolution. By integrating phylogenetic approaches, evolutionary rate estimations, and ERC analyses, we tested several key hypotheses: (1) mt genes produce phylogenies that are more concordant with those inferred from N-mt genes than with trees derived from random nuclear genes, reflecting shared phylogenetic signals driven by coevolution; (2) mt and N-mt genes exhibit a strong ERC within *Bombus*; (3) N-mt genes show higher *d_N_*/*d_S_* ratios compared to random nuclear genes, as predicted by the nuclear compensation hypothesis; (4) N-mt proteins that physically interact with mt proteins exhibit elevated *d_N_*/*d_S_* ratios and stronger ERC compared to N-mt proteins without such interactions; and (5) environmental factors influence mitonuclear coevolution in bumblebees, given the crucial role of mtDNA in adaptation.

## Results

### Mt and N-mt Genes Share Phylogenetic Signal

**Fig. 2.**
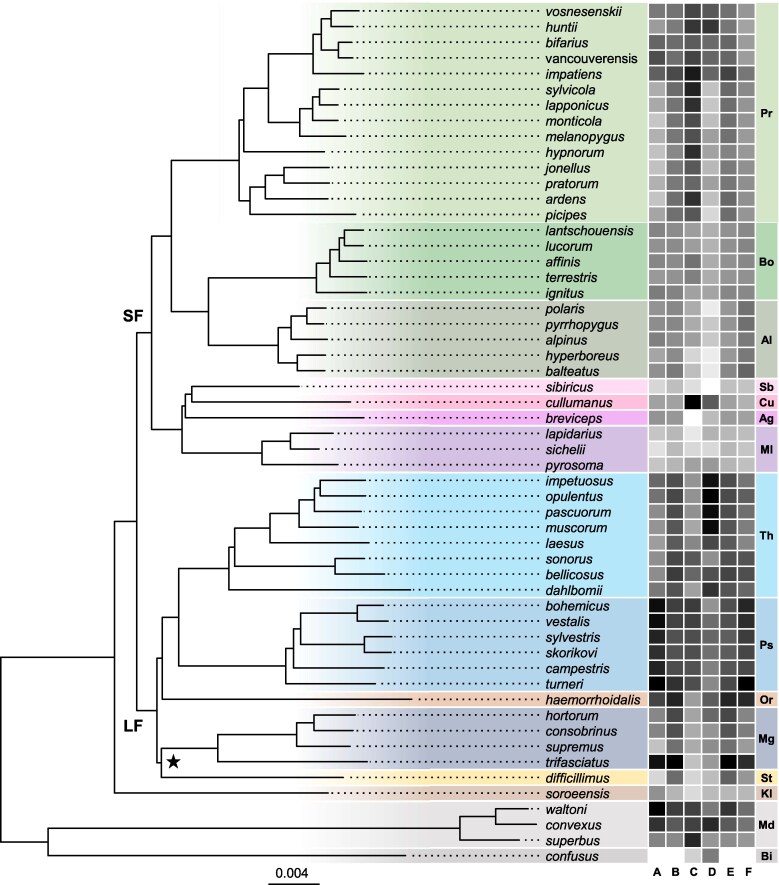
Bumblebee species tree and evolutionary rate heatmap. The tree was inferred using ASTRAL with input trees derived from RAxML analyses of individual UCE loci, and the topology was rescaled in RAxML so that branch lengths are proportional to substitutions per site. A star marks the single node with low local PP (0.40). SF and LF designate the “short-faced” and “long-faced” bumblebee clades, respectively. Clade colors correspond to *Bombus* subgenera: Pr, *Pyrobombus*; Bo, *Bombus*; Al, *Alpinobombus*; Sb, *Sibiricobombus*; Cu, *Cullumanobombus*; Ag, *Alpigenobombus*; Ml, *Melanobombus*; Th, *Thoracobombus*; Ps, *Psithyrus*; Or, *Orientalibombus*; Mg, *Megabombus*; St, *Subterraneobombus*; Kl, *Kallobombus*; Md, *Mendacibombus*; Bi, *Bombias*. On the right, a heatmap displays scaled branch length values from each gene dataset used in ERC analyses: a) mt, b) N-mt, c) glycolysis, d) random nuclear genes, e) N-mt genes encoding proteins that directly contact mt proteins, and f) N-mt genes encoding proteins that do not contact mt proteins. The heatmap is color-coded from white (low) to black (high).

Obtaining a robust species tree was a critical first step for our downstream analyses. Thus, using a dataset of 55 bumblebee genome sequences representing all 15 subgenera (Table 1; supplementary table S1, Supplementary Material online), we constructed a coalescent-based species tree with UCE loci. The resulting topology was largely well-resolved, with all subgenera forming monophyletic groups (Fig. 2). Most bumblebee subgenera were assigned to one of two main clades, the so-called long-faced and short-faced clades, which correspond broadly to differences in tongue length and head morphology (Cameron et al. 2007). However, one node had low local posterior probability (PP = 0.40), concerning the placement of subgenus *Subterraneobombus* (here represented by a single species, *Bombus difficillimus*), which formed a polytomy with *Megabombus* and a clade comprising the remaining long-faced subgenera (*Psithyrus*, *Thoracobombus*, and *Orientalibombus*). The ML trees inferred from concatenated amino acid alignments of gene datasets were marked by low node supports across subgeneric relationships when compared to this species tree (supplementary figs. S1 to S4, Supplementary Material online).

**Table 1:** Species and datasets analyzed in this study

Subgenus	Species	Dataset reference
*Alpigenobombus*	*Bombus breviceps*	Sun et al. (2021)
*Alpinobombus*	*Bombus alpinus*	Liu et al. (2024)
	*Bombus balteatus*	Christmas et al. (2021)
	*Bombus hyperboreus*	Liu et al. (2024)
	*Bombus polaris*	Sun et al. (2021)
	*Bombus pyrrhopygus*	Liu et al. (2024)
*Bombias*	*Bombus confusus*	Sun et al. (2021)
*Bombus*	*Bombus affinis*	Koch et al. (2023)
	*Bombus ignitus*	Sun et al. (2021)
	*Bombus lantschouensis*	Zhao et al. (2021)
	*Bombus lucorum*	DToL*
	*Bombus terrestris*	Crowley et al. (2023b)
*Cullumanobombus*	*Bombus cullumanus*	Sun et al. (2021)
*Kallobombus*	*Bombus soroeensis*	Sun et al. (2021)
*Megabombus*	*Bombus consobrinus*	Sun et al. (2021)
	*Bombus hortorum*	Crowley et al. (2021)
	*Bombus supremus*	Zhao et al. (2021)
	*Bombus trifasciatus*	Zhao et al. (2021)
*Melanobombus*	*Bombus lapidarius*	Cobb et al. (2024)
	*Bombus pyrosoma*	Sun et al. (2021)
	*Bombus sichelii*	Zhao et al. (2021)
*Mendacibombus*	*Bombus convexus*	Zhao et al. (2021)
	*Bombus superbus*	Sun et al. (2021)
	*Bombus waltoni*	Sun et al. (2021)
*Orientalibombus*	*Bombus haemorrhoidalis*	Sun et al. (2021)
*Psithyrus*	*Bombus bohemicus*	Zhao et al. (2021)
	*Bombus campestris*	Crowley et al. (2023f)
	*Bombus skorikovi*	Sun et al. (2021)
	*Bombus sylvestris*	Crowley et al. (2023e)
	*Bombus turneri*	Sun et al. (2021)
	*Bombus vestalis*	Crowley et al. (2025)
*Pyrobombus*	*Bombus ardens ardens*	Jeong et al. (2023)
	*Bombus bifarius*	Heraghty et al. (2020)
	*Bombus huntii*	Koch et al. (2024)
	*Bombus hypnorum*	Crowley et al. (2023a)
	*Bombus impatiens*	Sadd et al. (2015)
	*Bombus jonellus*	Broad et al. (2025)*
	*Bombus lapponicus*	Liu et al. (2024)
	*Bombus melanopygus*	Wham et al. (2021)
	*Bombus monticola*	DToL*
	*Bombus picipes*	Sun et al. (2021)
	*Bombus pratorum*	Crowley et al. (2023c)
	*Bombus sylvicola*	Christmas et al. (2021)
	*Bombus vosnesenskii*	CCGP*
	*Bombus vancouverensis nearcticus*	Heraghty et al. (2020)
*Sibiricobombus*	*Bombus sibiricus*	Sun et al. (2021)
*Subterraneobombus*	*Bombus difficillimus*	Sun et al. (2021)
*Thoracobombus*	*Bombus bellicosus*	Gonçalves et al. (2025)
	*Bombus dahlbomii*	Martínez et al. (2024)
	*Bombus impetuosus*	Zhao et al. (2021)
	*Bombus laesus*	Zhao et al. (2021)
	*Bombus muscorum*	Broad et al. (2024)
	*Bombus opulentus*	Sun et al. (2021)
	*Bombus pascuorum*	Crowley et al. (2023d)
	*Bombus sonorus*	CCGP*
*Outgroup*	*Apis mellifera*	Wallberg et al. (2019)

Additional details, including accession numbers, are provided in supplementary table S1, Supplementary Material online. Datasets marked with an asterisk were generated by The Darwin Tree of Life Project Consortium (2022) (DToL) and by The California Conservation Genomics Project (CCGP) (Shaffer et al. 2022) but lack an associated publication.

**Fig. 3.**
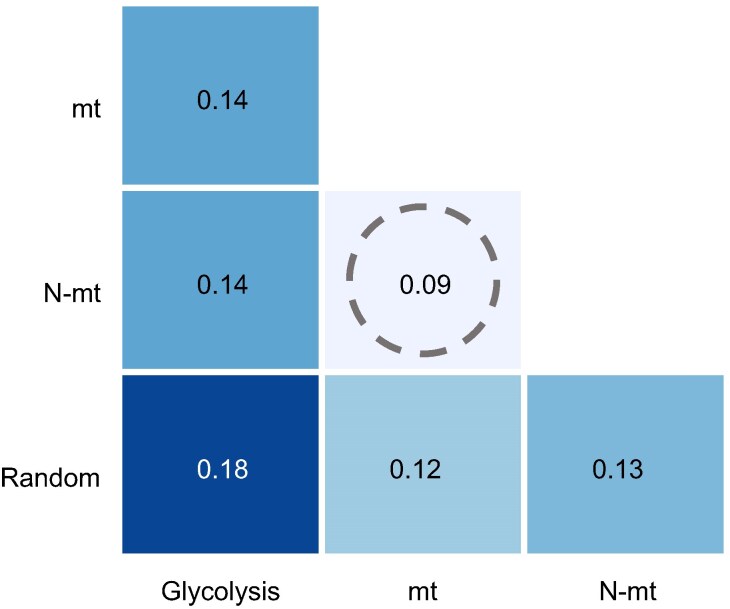
Tree topology incongruences quantified using the generalized Robinson–Foulds metric (Smith 2020). Values range from 0 (full concordance) to 1 (full discordance). The heatmap shows that the topologies of the mt and N-mt trees align more closely with each other than with trees inferred using glycolysis genes or random orthologs. This emphasizes the strong evolutionary signal shared between mt and N-mt genes (highlighted with the dotted circle).

We also tested whether mt and N-mt gene trees showed greater similarity when compared to trees derived from random nuclear genes. To quantify the concordance between phylogenetic trees inferred from different datasets, we used the generalized Robinson–Foulds metric to measure topological incongruence across the best ML trees. Values can range from 0, indicating identical topologies, to 1, representing complete discordance (Smith 2022). Comparisons between trees revealed that the N-mt tree exhibited the highest topological congruence with the mt tree (Fig. 3), whereas the topology of both the mt and N-mt trees was less congruent with trees constructed using glycolysis genes and random orthologs (Fig. 3). These findings suggest that the phylogenetic signal of mt genes is more aligned with that of N-mt genes than with genes from other pathways or random orthologs.

### Mt and N-mt Genes Evolve Faster Than Nuclear Genes

We found that bumblebee mt genes displayed markedly higher rates of amino acid substitutions compared to nuclear genes, ranging from 2.4 to 4.3 times faster than N-mt genes and 11.4 to 21.2 times faster than random nuclear orthologs (supplementary table S2, Supplementary Material online). Furthermore, the rate of mt amino acid substitutions varied among species (Fig. 2), with the fastest-evolving species (*Bombus [Psithyrus] turneri*) showing rates approximately 1.8 times higher than the slowest-evolving species (*Bombus [Bombias] confusus*). N-mt genes exhibited, on average, amino acid substitution rates around 6.1 times faster than random orthologs and 9.5 times faster than glycolysis genes. Among control protein datasets, we found that glycolysis genes showed the slowest amino acid substitution rates (supplementary table S2, Supplementary Material online).

**Fig. 4.**
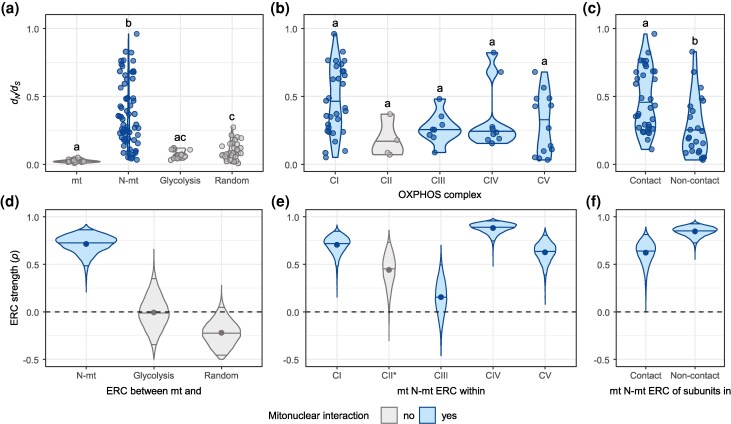
(a to c) *d_N_*/*d_S_* ratios of mt and nuclear gene sets. In each panel, the mean *d_N_*/*d_S_* ratio of groups that do not share a letter are considered statistically significant from Dunn's test after Bonferroni correction for multiple comparisons. (a) Comparison of *d_N_*/*d_S_* ratios of mt genes, N-mt genes, and nuclear-encoded genes that do not interact with mt proteins (glycolysis genes and random orthologs). (b) Within-complex *d_N_*/*d_S_* ratios of N-mt genes. (c) Comparison of *d_N_*/*d_S_* ratios of N-mt genes that contain or lack residues that directly contact mt residues. (d to f) ERCs among different datasets. The distribution of 10,000 bootstrap replicates of the correlation coefficient (*ρ*) are shown. Dot is the mean, central black bar is the median, top and bottom black bars are the 95% CI. (d) ERC among mt and nuclear-encoded genes. (e) ERC among mt and N-mt genes, split by OXPHOS complex. * indicates CII correlation is between nuclear-encoded CII genes and all mt genes. (f) ERC among mt and N-mt genes that contain or lack residues that directly contact mt residues.

Analyzing the distribution of *d_N_*/*d_S_* ratios, we observed that N-mt genes displayed higher *d_N_*/*d_S_* ratios compared to mt genes (*P* < 0.0001), glycolysis genes (*P* < 0.0001), and random nuclear orthologs (*P* < 0.0001). Conversely, *d_N_*/*d_S_* values of mt genes were comparable to those of glycolysis genes, but differed from random orthologs (*P* = 0.006) (Fig. 4a). Within each OXPHOS complex, CI *d_N_*/*d_S_* ratios were the highest (median = 0.41) and CII ratios were the lowest (median = 0.17). However, no significant differences in *d_N_*/*d_S_* ratios were observed among complexes (Fig. 4b). Finally, N-mt genes with residues directly contacting mt-encoded proteins exhibited significantly higher *d_N_*/*d_S_* values (median = 0.42) compared to N-mt genes that do not contact (median = 0.19; *P*  *=* 0.0003; Fig. 4c). *d_N_*/*d_S_* ratios for individual genes are available as supplementary table S3, Supplementary Material online.

### Evolutionary Rates of mt and N-mt Genes Are Strongly Correlated

We normalized focal gene evolutionary rates against those of random nuclear genes that do not interact with mt products, controlling for lineage-specific factors that could drive rate covariation independently of coevolution. Moreover, we used phylogenetic independent contrasts (PICs) to control for statistical nonindependence among species. We found a positive and significant correlation between mt and N-mt evolutionary rates (*ρ* = 0.72, 95% confidence interval [CI, 0.47 to 0.86]; Fig. 4d). In contrast, no significant correlation was found between mt and random nuclear orthologs (*ρ* = −0.22, 95% CI [−0.46 to 0.08]; Fig. 4d) or mt and glycolysis genes (*ρ* = −0.01, 95% CI [−0.35 to 0.33]; Fig. 4d). When conducting ERC tests within subgenera to verify if the strength of correlation is lineage-specific, results revealed substantial variation in the magnitude of correlations, ranging from perfect positive associations (e.g. *Megabombus*, *ρ* = 1.00, *n* = 4) to more moderate relationships (e.g. *Pyrobombus*, *ρ* = 0.52, *n* = 14). However, these comparisons lacked statistical power due to the small number of species per subgenus. To mitigate this, we conducted the ERC considering the two major bumblebee clades, long-faced and short-faced (Fig. 2). A positive and significant correlation with similar magnitude was observed for both clades (short-faced: *ρ* = 0.55, 95% CI [0.13 to 0.79]; long-faced: *ρ* = 0.61, 95% CI [0.16 to 0.80]).

Subsetting the genes by their OXPHOS complexes (Fig. 4e), we identified strong positive correlations between N-mt and mt subunits of CI (*ρ* = 0.77, 95% CI [0.58 to 0.88]), CIV (*ρ* = 0.89, 95% CI [0.74 to 0.95]), and CV (*ρ* = 0.63, 95% CI [0.39 to 0.80]). Notably, mt and nuclear-encoded subunits of CIII showed weak and nonsignificant correlation (*ρ* = 0.16, 95% CI [−0.21 to 0.49]). Yet, comparing N-mt genes of CIII against all mt genes revealed a stronger and significant correlation (*ρ* = 0.69, 95% CI [0.50 to 0.80]). Since CII subunits are exclusively nuclear-encoded, no correlation was expected with mt genes. However, CII genes exhibited a moderate correlation with mt genes from other OXPHOS complexes (*ρ* = 0.45, 95% CI [0.27 to 0.70]; Fig. 4e).

Finally, we divided our N-mt dataset into subunits that directly contact mt subunits and those that do not. The evolutionary rates of contact N-mt subunits exhibited a lower correlation with mt rates (*ρ* = 0.64, 95% CI [0.35 to 0.82]) compared to noncontact subunits (*ρ* = 0.85, 95% CI [0.71 to 0.92]; Fig. 4f). Due to the overlapping 95% CI of these bootstrapped correlation coefficients, we concluded that the observed differences between contact and noncontact genes were not statistically significant. Correlation coefficients and 95% CI for all conducted ERC are available as supplementary table S4, Supplementary Material online.

### Bumblebee Species From Colder Environments Experience Faster mt Evolution

Given the role that mitonuclear interactions may play in local adaptation, we tested whether bumblebee mitonuclear coevolution is associated with the climatic niche of each species. To this end, we extracted environmental variables from the geographical coordinates of bumblebee sampling sites and modeled the association between these variables and evolutionary rates. To reduce multicollinearity among environmental variables, we performed a principal component analysis (PCA) on elevation, geographic coordinates (latitude and longitude), and five climatic variables. The first two principal components (PC1 and PC2) explained 73.45% of the total variance in the environmental data (PC1: 47.69%, PC2: 25.76%). PC1 was strongly associated with elevation, temperature annual range (bio7), and temperature seasonality (bio4), while PC2 was primarily influenced by maximum temperature of the warmest month (bio5) and latitude (supplementary table S5, Supplementary Material online). These components were used as predictors in subsequent analyses to examine their relationship with mitonuclear evolutionary rates.

**Fig. 5.**
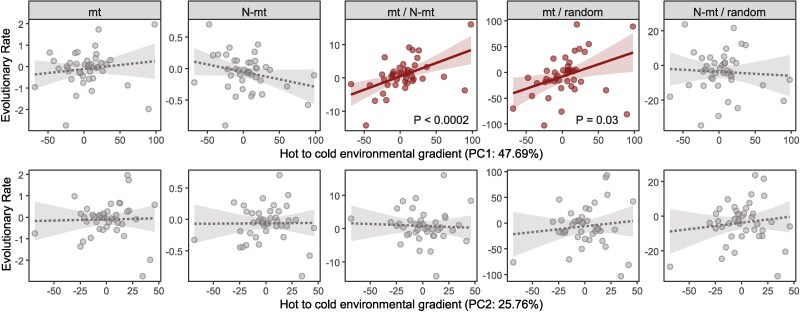
Relationships between environmental gradients (PC1 and PC2) and evolutionary rates. Scatterplots show the relationships between the first two principal components (PC1 and PC2) and different evolutionary rate measures across the bumblebee phylogeny. PC1 represents an environmental gradient where higher values indicate colder, high-elevation environments, and lower values correspond to warmer, lowland regions. PC2 represents a secondary temperature gradient, capturing seasonal temperature extremes, with lower values associated with regions experiencing warmer summers. Each panel displays the evolutionary rate of a specific dataset as a function of PC1 (top) or PC2 (bottom). Regression lines were fitted using a linear model. Illustrations in red, with solid lines, indicate significant relationships, while those in gray, with dashed lines, indicate nonsignificant relationships. PC2 values were multiplied by −1 to maintain consistency with PC1 (hot-to-cold environmental gradient).

A multivariate analysis of variance (MANOVA) revealed a significant effect of PC1 on evolutionary rates (Pillai's trace = 0.486, *P* = 0.00015), whereas PC2 had no significant effect (Pillai's trace = 0.125, *P* = 0.41). In follow-up univariate analyses, PC1 was positively associated with the mt/N-mt ratio (*β* = 0.086, *P* = 0.0002, explaining 26.2% of the variance) and the mt/random gene ratio (*β* = 0.426, *P* = 0.03, explaining 8.6% of the variance) (Fig. 5; supplementary table S6, Supplementary Material online). This indicates that bumblebee species from colder, high-elevation environments exhibit faster mt evolutionary rates relative to both N-mt genes and the overall nuclear genome. No significant associations were found between climatic variables and the remaining evolutionary metrics tested (mt and N-mt rates, N-mt/random gene ratio; Fig. 5; supplementary table S6, Supplementary Material online). To further validate these findings, we compared mt/N-mt and mt/random ratios between species at the environmental extremes of the PC1 gradient (lowest and highest 33%). Consistent with the other results, species from colder environments (high PC1) showed significantly higher mt/N-mt and mt/random ratios than those from warmer environments (low PC1) (Wilcoxon rank-sum test, *P* = 0.02 and 0.04, respectively).

## Discussion

### Mitonuclear Phylogenetic Signal in a Genus-Level Framework

We detected a strong positive correlation between the evolutionary rates of mt and N-mt genes in bumblebees (*ρ* = 0.72), whereas nuclear gene sets lacking functional interaction with mt products exhibited weak or negligible correlation (Fig. 4d). These findings support a fundamental prediction of mitonuclear coevolution—that coordinated rate variation between mt and N-mt genes help maintain mt function by preserving mitonuclear interactions, while such covariation is absent between mt and noninteracting nuclear genes. Similar results have been observed in other taxa such as bivalves (Piccinini et al. 2021), vertebrates (Zhang and Broughton 2013; Weaver et al. 2022), and various insect orders including Hymenoptera (Li et al. 2017; Yan et al. 2019). However, unlike prior studies that sampled a broad range of deeply divergent lineages, our investigation focused on a single genus, offering novel insights on mitonuclear coevolution among closely related species and over shorter evolutionary times.

Previous studies have suggested that N-mt genes often exhibit a phylogenetic signal more similar to mt genes than with other nuclear genes (Sloan et al. 2017; Piccinini et al. 2021), a trend confirmed in our dataset (Fig. 3). In our best ML trees, mt and N-mt gene tree topologies were highly similar, whereas trees inferred from other gene sets showed greater incongruence (Fig. 3). This finding aligns with long-standing challenges in resolving the bumblebee tree of life, even with complete mt genomes (Gonçalves et al. 2024) or thousands of nuclear loci (Sun et al. 2021). These difficulties arise from extensive gene tree discordance, likely driven by the recent diversification of bumblebees and high levels of incomplete lineage sorting (Cameron et al. 2007; Sun et al. 2021). Nevertheless, our results demonstrate that mt and N-mt genes share a strong phylogenetic signal. While reconstructing bumblebee phylogenetic relationships was not our primary goal, our approach integrating UCE loci and coalescence-based analyses yielded a well-supported species tree (Fig. 2), highlighting a promising avenue for future studies in bumblebee systematics.

Our results further indicate that the strength of mitonuclear ERC is uniform across lineages, at least when comparing the two major bumblebee clades. This contrasts with patterns observed in other taxa, where mitonuclear ERC strength varies among lineages. For instance, in mammals, ERC is stronger in primates than in rodents or carnivores (Weaver et al. 2022), while among insects, Hymenoptera show stronger ERC than Diptera (Yan et al. 2019). Such differences suggest variability in the tightness of mitonuclear adaptation and have been linked to various factors such as differences in metabolic demands (Osada and Akashi 2012), ecological niches (Trier et al. 2014), and life history traits (Roux et al. 2014; Camus et al. 2020). Given the variability in mt substitution rates across bumblebee lineages, as observed here and in prior studies (Lin et al. 2019; Gonçalves et al. 2024), we expected stronger lineage-specific ERC signals. The absence of such patterns may reflect limited resolution due to our sampling of single individuals per species, as finer-scale ERC signals may be more apparent at the population level (Barreto et al. 2018).

### Shifts in Selection Constraints Underlying Mitonuclear Coevolution

A leading hypothesis explaining how mt and nuclear genomes maintain coadaptation over evolutionary time is nuclear compensation, whereby N-mt genes compensate for deleterious mutations arising from mutational erosion of mt genes (Osada and Akashi 2012). This hypothesis predicts that N-mt genes should exhibit relatively intensified positive selection compared to random nuclear genes, reflecting the compensatory mechanism (Nabholz et al. 2013; Havird and Sloan 2016). Our findings support this prediction, as evidenced by significantly higher *d_N_*/*d_S_* ratios in N-mt genes compared to control nuclear genes (Fig. 4a), also consistent with previous observations in Hymenoptera (Gibson et al. 2010; Li et al. 2017). Furthermore, when we accounted for protein–protein contact, N-mt proteins that directly interact with mt proteins exhibited higher *d_N_*/*d_S_* ratios than those that do not (Fig. 4c), reinforcing the notion of compensatory evolution.

When we restricted our ERC and *d_N_*/*d_S_* analyses to comparisons within individual OXPHOS complexes, intriguing results emerged. CI, CIV, and CV showed high *d_N_*/*d_S_* ratios in their N-mt genes (Fig. 4b) along with robust correlations between their N-mt and mt subunits (Fig. 4e), supporting a scenario of tight mitonuclear coevolution. However, CIII showed a weak correlation between its mt and N-mt subunits (*ρ* = 0.16). We suspected that this was biased since CIII has only one mt-encoded gene (Cyt *b*). With only one mt gene to capture the evolutionary dynamics of the entire complex, there is less statistical power and potentially distinct selection pressures, thereby limiting the observable correlation. Indeed, when we calculated the ERC between N-mt genes of CIII and all mt genes, a stronger correlation was observed (*ρ* = 0.69), suggesting that although *Cyt b* may not fully capture the coevolutionary signal, N-mt genes in CIII still share a broader coevolutionary signal with the mt genome.

CII exhibited the lowest *d_N_*/*d_S_* ratios among the complexes (although not statistically significant), aligning with a prediction of mitonuclear coevolution. CII is exclusively formed by N-mt genes and should not be subject to the nuclear compensation effect that increases *d_N_*/*d_S_* ratios in mt-interacting genes (Barreto et al. 2018; Weaver et al. 2020; Piccinini et al. 2021). However, our ERC results revealed a moderate yet significant correlation between CII and mt genes from other complexes (*ρ* = 0.45), which goes against expectations. A possible explanation for these results could be a relaxed purifying selection is operating in these genes, particularly in succinate dehydrogenase subunit D (SDHD), which exhibited *d_N_*/*d_S_* ratios twice as high as the median (SDHD *d_N_*/*d_S_* = 0.37; median *d_N_*/*d_S_* of CII genes = 0.17), hinting at complex selective dynamics beyond simple nuclear compensation.

### Mitonuclear Coevolution and Local Adaptation of Bumblebees

When considering environmental variables, our findings suggested a potential link between mitonuclear evolutionary dynamics and climatic niche adaptation in bumblebees. We found that species from colder, high-elevation environments exhibited increased mt evolutionary rates relative to both N-mt genes and random nuclear genes (Fig. 5). This aligns with previous studies documenting accelerated mt evolution in high-altitude arthropods, often attributed to adaptive responses to hypoxia and low temperatures (Zhang et al. 2013; Li et al. 2018; Yuan et al. 2018). However, absolute mt evolutionary rates did not show a significant association with climatic variation (*P* = 0.45), suggesting that the environment may be shaping evolutionary dynamics in both genomes, but with a stronger effect on the mt genome. Comparisons of mt/random and N-mt/random rate ratios suggest that the observed increase in the mt/N-mt ratio in colder environments is driven by mt rate acceleration (mt/random ratio: *β* = 0.43, *P* = 0.03), rather than a deceleration of N-mt rates (N-mt/random ratio: *P* = 0.43; absolute N-mt rates: *P* = 0.06). Therefore, the observed effect implies targeted mtDNA divergence in response to environmental pressures rather than a uniform shift across both genomes.

Several evolutionary processes could underlie these findings. High-altitude environments are often associated with smaller *N_e_* values, which can lead to the fixation of slightly deleterious mutations in mt genomes due to increased genetic drift (Iverson et al. 2025), accelerating mt evolutionary rates. In bumblebees, montane species have been shown to exhibit reduced *N_e_* (Liu et al. 2024; Lozier et al. 2023), and populations at higher elevations often show lower genetic diversity and reduced gene flow compared to lowland counterparts (Lozier et al. 2011, 2021; Han et al. 2014; Jackson et al. 2018; Heraghty et al. 2022). Additionally, selection pressures linked to temperature and oxygen availability may favor specific mt variants that optimize cellular respiration under cold and hypoxic conditions. In ectothermic organisms, mtDNA variation has been shown to influence metabolic efficiency and thermal tolerance, contributing to local adaptation (Willett 2011; Pichaud et al. 2013; Camus et al. 2017). While previous transcriptomic studies in bumblebees have shown differential expression of OXPHOS and energy metabolism genes in response to cold and hypoxic environments (Liu, Jin et al. 2020; Liu, Zhao et al. 2020; Liang et al. 2022), our findings suggest that mitonuclear interactions themselves may contribute to enhanced fitness at high altitude, pointing to an unnoticed mechanism of local adaptation in this group.

A key consequence of an increased mt/N-mt evolutionary rate ratio is potential disruption of mitonuclear coevolution, as faster mt gene evolution relative to N-mt genes could lead to functional mismatches. In bumblebees, maintaining efficient energy production is particularly critical, as they rely on pre-flight thermogenesis to warm flight muscles at low temperatures. Unlike many other insects, the buff-tailed bumblebee (*B. terrestris*) employs an mt glycerol-3-phosphate dehydrogenase pathway to facilitate thermogenesis through increased mt uncoupling (Masson et al. 2017). If mt uncoupling enhances cold tolerance by promoting heat generation, relaxed mitonuclear constraints in high-altitude species might reflect an adaptive response, where reduced coupling efficiency helps sustain metabolic activity under low temperatures. In vertebrates, similar mechanisms occur through mt uncoupling proteins that facilitate proton leak, enabling non-shivering thermogenesis (Ricquier 2011; Nicholls 2021).

Despite these implications, our study is limited by the use of a single individual per species, oversimplifying intraspecific variation. Since bumblebees often occupy broad and environmentally heterogeneous geographic ranges, further studies are needed to test how generalizable these patterns are across their full distributions. Moreover, some bumblebee species exhibit heteroplasmy (Françoso et al. 2016; Ricardo et al. 2020 ), which violates the clonal and haploid nature of mtDNA, thus future research should also focus on understanding its consequences on the results we observed. If the same set of N-mt genes must interact with products from different mt genomes within an individual, the purging of deleterious alleles could become less effective, potentially impacting fitness (Sharpley et al. 2012; McCauley 2013; Meshnik et al. 2022). Research integrating population-level genomic data with functional assays will be crucial for disentangling the adaptive significance of mitonuclear evolution in bumblebees and the role of heteroplasmy in mitonuclear interactions.

## Conclusion

Here we provide strong evidence for mitonuclear coevolution in bumblebees, as indicated by a significant ERC between mt and N-mt genes but not between mt and random nuclear genes, supporting the hypothesis that mitonuclear interactions drive ERCs even at the genus level. The consistency of ERC strength across bumblebee lineages suggests that mitonuclear coevolution is a pervasive feature of their evolution, distinct from findings in other taxa. Moreover, species from colder environments exhibit elevated mt evolutionary rates, hinting at a potential link between mitonuclear evolution and thermal adaptation. These results highlight bumblebees as an excellent model for studying mitonuclear coevolution and its role in environmental adaptation. With expanding genomic resources and advances in functional genomics, this study lays the foundation for deeper investigations into the mechanisms driving mitonuclear coevolution and its broader evolutionary significance.

## Methods

### Genomic Dataset

We downloaded from GenBank the genomic data of 36 bumblebee species and *Apis mellifera*. We also retrieved Illumina and PacBio datasets of 19 additional bumblebee species without an available assembly from the NCBI Sequence Read Archive (SRA) (Table 1; supplementary table S1, Supplementary Material online). We adopted a strategy to use these reads by mapping them to the genome of a species belonging to the same subgenus for which a high-quality genome assembly was available (supplementary table S1, Supplementary Material online). Illumina reads were processed using Trimmomatic v0.39 (Bolger et al. 2014) to remove adapter sequences, and reads were mapped using BWA-MEM v0.7.17 (Li 2013). PacBio reads were processed using Cutadapt v4.4 (Martin 2011) to remove adapter sequences and were subsequently mapped against the reference genomes using Minimap2 v2.24 (Li 2018) with the preset designed for PacBio HiFi genomic reads (-x map-pb). We employed Samtools v1.16 (Li et al. 2009) to extract consensus sequences for both types of datasets by calling the most frequent base (-m simple).

### Sequence Retrieval

We obtained nucleotide sequences of *B. terrestris* and *A. mellifera* for 13 mt OXPHOS genes and 65 N-mt OXPHOS genes from the Kyoto Encyclopedia of Genes and Genomes (KEGG; Kanehisa 2000) (supplementary table S3, Supplementary Material online). Using *B. terrestris* sequences as queries, BLASTn searches were conducted to retrieve coding sequences from other bumblebee species. Redundant and incomplete sequences were visually identified and eliminated, retaining only full-length coding sequences for further analysis. Additionally, two control datasets were compiled. The first control dataset consisted of 14 glycolysis genes retrieved from KEGG and extracted from the genomes following the same procedure applied to N-mt OXPHOS genes. These genes were chosen because they are involved in essential energy metabolism processes in the cell without interacting with mt proteins. The second control dataset comprised 30 randomly selected single-copy orthologs not involved in mt function obtained using the Hymenoptera dataset of BUSCO v5.6.1 (Manni et al. 2021).

Nuclear-encoded proteins that function in mitochondria contain an N-terminal motif (target peptide) that is cleaved upon mt entry. As they are not involved in mitonuclear interactions, these mt-targeting sequences may bias the comparative analysis of evolutionary rates for these proteins. Therefore, we identified mt-targeting cleavage sites using TargetP v2.0 (Armenteros et al. 2019) and excluded the predicted targeting motifs from our final dataset.

We aligned all sequences using the codon-aware program MACSE v2.03 (Ranwez et al. 2018), which preserves the reading frame of coding sequences. Ambiguously aligned fragments were trimmed using Gblocks v0.91b (Talavera and Castresana 2007) using reduced stringency settings (-b5 = h).

### Phylogenetic Inference

To obtain a reference species tree for downstream analyses, we extracted UCE loci from the retrieved genomes. We followed the standard pipeline of Phyluce v1.7.3 (Faircloth 2016) to identify and extract UCE loci by aligning the principal hymenopteran v2 probe set (Branstetter et al. 2017) to the genomes and by slicing out 500 bp of flanking sequences from target loci. Sequences were aligned using MAFFT v7.130b (Katoh et al. 2019) and trimmed with Gblocks. Finally, alignments were filtered to exclude loci with less than 75% of the taxa present. The resulting alignment set included 1,608 loci and 1,732,583 bp of sequence data, of which 270,905 were informative.

Because of the reported high levels of gene tree discordance in bumblebee phylogenomic datasets (Sun et al. 2021), we employed ASTRAL-III v5.7.1 (Zhang et al. 2018) to infer a coalescent-based species tree. Maximum Likelihood (ML) gene trees for each UCE locus were estimated using RAxML v8.2.12 (Stamatakis 2014) under the GTR + G + I model, which was assumed following Abadi et al. (2019). Branch support was measured by 1,000 bootstrap replicates. For each locus, the best tree was retained as input for the ASTRAL analysis. Since ASTRAL estimates branch lengths in coalescent units and only for internal branches, we ran RAxML to obtain branch lengths proportional to substitutions per site. We used a concatenated alignment of the UCE loci as input and the same parameters described above, but setting the ASTRAL tree as a topological constraint.

We also inferred unconstrained phylogenies from concatenated amino acid datasets to check for convergent phylogenetic signals across mt and N-mt genes. Similarly, phylogenies were inferred from control datasets to gain insights of the phylogenetic signal of non-mt-interacting genes. Trees were built using RAxML with the same parameters described above, but using the automated protein model assignment algorithm and a gamma model of rate heterogeneity (-m PROTGAMMAAUTO).

To compare the topologies of the resulting trees, pairwise generalized Robinson–Foulds distances (Smith 2020) were calculated for the best resulting ML tree of each dataset using the TreeDistance() function in R package TreeDist (Smith 2022). These distances quantify topological dissimilarity between pairs of trees by comparing their constituent bipartitions (splits), applying a similarity score based on information theory. In this framework, a distance of 0 indicates identical topologies, while larger values reflect greater dissimilarity in the splits between trees. Thus, lower distances signify greater topological similarity.

### Evolutionary Rates

We inferred the evolutionary rates of each set of concatenated amino acid sequence alignments (mt, N-mt, glycolysis, and random orthologs) by estimating branch lengths constrained on the coalescent-based species tree. This approach ensured that the topology represented bumblebee species relationships and was not re-estimated based on input sequence data. We used *A. mellifera* as the outgroup in phylogenetic analyses but did not include it in ERC analyses. For nuclear genes, we employed the automated protein model assignment algorithm of RAxML (-m PROTGAMMAAUTO), while for mt genes we used a substitution model tailored for mt proteins of arthropods (-m PROTGAMMAIMTART; Abascal et al. 2006). Branch support was assessed via 1,000 bootstrap replicates. Root-to-tip branch lengths were extracted using the distRoot function in R package adephylo (Jombart et al. 2010). Branch lengths were also estimated individually for each OXPHOS complex, based on concatenated mt and N-mt amino acid alignments.

To gain insights into the selective pressures acting on the genes, we estimated the synonymous and nonsynonymous substitution rates from nucleotide sequence alignments of individual genes using codeml in PAML v4.9 (Yang 2007). This involved fitting a model in which *d_N_*/*d_S_* is assumed to be constant across all sites and branches (model = 0, NSsites = 0). To assess variations in *d_N_*/*d_S_* across datasets, we employed a Kruskal–Wallis test followed by a *post hoc* Dunn's test with Bonferroni correction for multiple comparisons in R (R Core Team 2021).

### ERC Analyses

One potential challenge in ERC analyses arises from demographic factors that may lead to autocorrelated evolutionary rates among gene sets not undergoing coevolution. Moreover, species are statistically nonindependent due to shared evolutionary history, which can introduce spurious correlations across traits. This concern is particularly relevant when analyzing closely related, congeneric species, as in this study. To address these issues, we used two complementary approaches. First, we normalized focal gene branch lengths against those of random nuclear genes that do not interact with mt products, controlling for lineage-specific factors that could drive rate covariation independently of coevolution. Then, we computed PICs using the R package ape (Paradis and Schliep 2019), with the rescaled ASTRAL topology as the reference tree, to account for the nonindependence of species due to phylogenetic relatedness. This step was made to address the nonindependence of species caused by their relatedness on a phylogeny.

To estimate ERCs, Pearson's correlation tests were conducted on the computed PICs of mt-encoded and nuclear-encoded gene sets. To assess within-complex mitonuclear correlation, correlation analyses were also performed on PICs estimated from subsets of genes belonging to each OXPHOS complex. We also divided our datasets across different bumblebee lineages (subgenera and long-faced/short-faced clades) to test if ERC strength is lineage-specific. Finally, to elucidate the potential impact of protein–protein contact on mitonuclear coevolution dynamics, we partitioned our N-mt dataset into groups of subunits predicted to interact physically with mt-encoded subunits and those lacking any direct interaction with mt proteins (Weaver et al. 2022). These subsets were then correlated with the mt dataset. All correlation tests were conducted using the corr.test function available in R.

To statistically evaluate differences in correlations within our gene sets, we conducted bootstrapping on the resulting Pearson's correlation coefficients (*ρ*). This involved generating 10,000 iterations for each comparison using the boot function in R. Subsequently, we calculated 95% CI from the resulting distributions using the boot.ci function in R, employing the adjusted bootstrap percentile (BCa) method. If the 95% CI included zero, we considered the correlation coefficient nonsignificant at a significance level of 0.05.

### Environmental Variables

We examined the association between environmental variables and bumblebee mitonuclear evolution using a MANOVA. Geographic coordinates and elevation (in meters) of the bumblebee sampling sites were obtained from the corresponding NCBI BioSample metadata associated with each analyzed genome (supplementary table S1, Supplementary Material online). These geographical coordinates were utilized to extract five additional climatic variables from the WorldClim database (Fick and Hijmans 2017) for each data point: mean annual temperature (Bio1), temperature seasonality (Bio4; expressed as standard deviation multiplied by 100), maximum temperature of the warmest month (Bio5), minimum temperature of the coldest month (Bio6), and temperature annual range (Bio7; the difference between Bio5 and Bio6). Previous studies have identified these variables as crucial in defining the ranges of bumblebee species (Dellicour et al. 2017; Sirois-Delisle and Kerr 2018; Françoso et al. 2019; Naeem et al. 2019; Krechemer and Marchioro 2020).

Climatic variables were extracted using the R package raster (Hijmans 2023). To control for environmental variable interdependence (e.g. elevation and mean annual temperature), we performed a PCA using standardized values of environmental predictors. This approach allowed us to reduce dimensionality while retaining key axes of environmental variation. To account for phylogenetic relatedness, we computed PICs for both the principal components and the evolutionary rates. Because our aim was to examine associations between absolute evolutionary rates and climate, rather than test for coevolution, we used unnormalized evolutionary rates. A MANOVA was run on PIC-transformed data, with mt evolutionary rates, N-mt evolutionary rates, mt/N-mt rate ratio, mt/random genes rate ratio, and N-mt/random genes rate ratio as dependent variables. PC1 and PC2 were included as independent variables. Statistical significance was assessed using *P*-values from the univariate linear models within the multivariate framework. Finally, to complement the analyses and address potential concerns about model assumptions, we also compared species groups at the environmental extremes of the PC1 gradient. Specifically, we selected the 33% of species with the lowest and highest PC1 scores and compared their evolutionary rates using a Wilcoxon rank-sum test. All analyses were performed in R.

## Supplementary Material

evaf123_Supplementary_Data

## Data Availability

Scripts and workflows are available at a GitHub repository: https://github.com/leonardotgoncalves/mitonuclear_bombus. Additional data files supporting our findings (e.g. phylogenetic trees, sequence alignments) are available on Zenodo: https://doi.org/10.5281/zenodo.15271492.
