# Supplementary material for: Mitonuclear Coevolution in Bumblebees (Bombus): Genomic Signatures and Its Role in Climatic Niche Adaptation
Source: Genome Biol Evol. 2025 Jun 13;17(7):evaf123. doi: 10.1093/gbe/evaf123 (PMC12223992; doi:10.1093/gbe/evaf123)
Supplement: evaf123_Supplementary_Data [file evaf123_supplementary_data.zip › Supplementary Figures.pdf]

Supplementary Figures

Mitonuclear coevolution in bumblebees (*Bombus*): genomic signatures and its role in climatic niche adaptation

Leonardo Tresoldi Gonçalves, Pedro Henrique Pezzi, Maríndia Deprá, Elaine Françoso

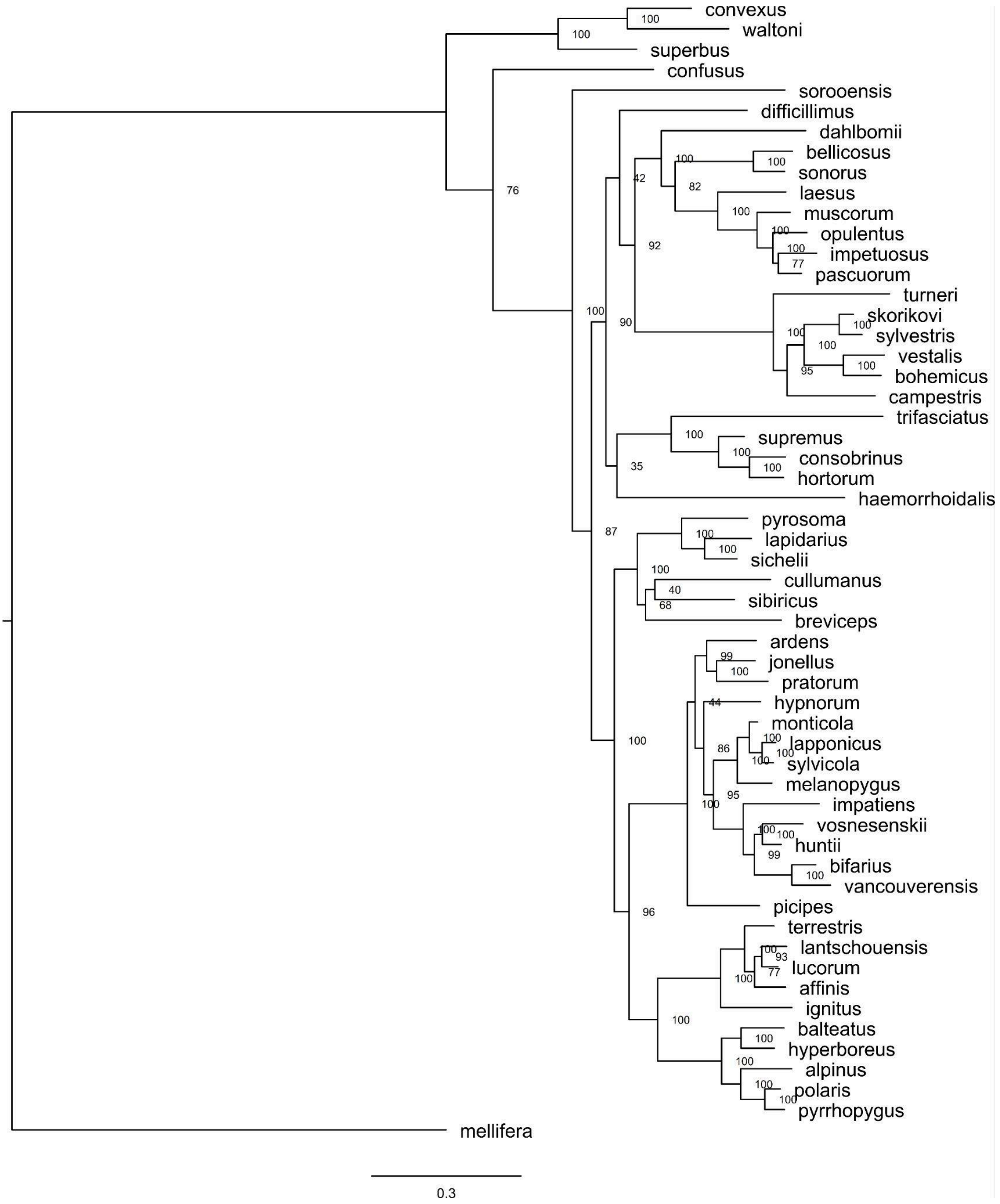

Supplementary figure 1. Unconstrained Maximum Likelihood phylogeny built using the concatenated amino acid matrix of mt genes. Scale bar indicates estimated substitutions per site. Only bootstrap values above 70 are shown.

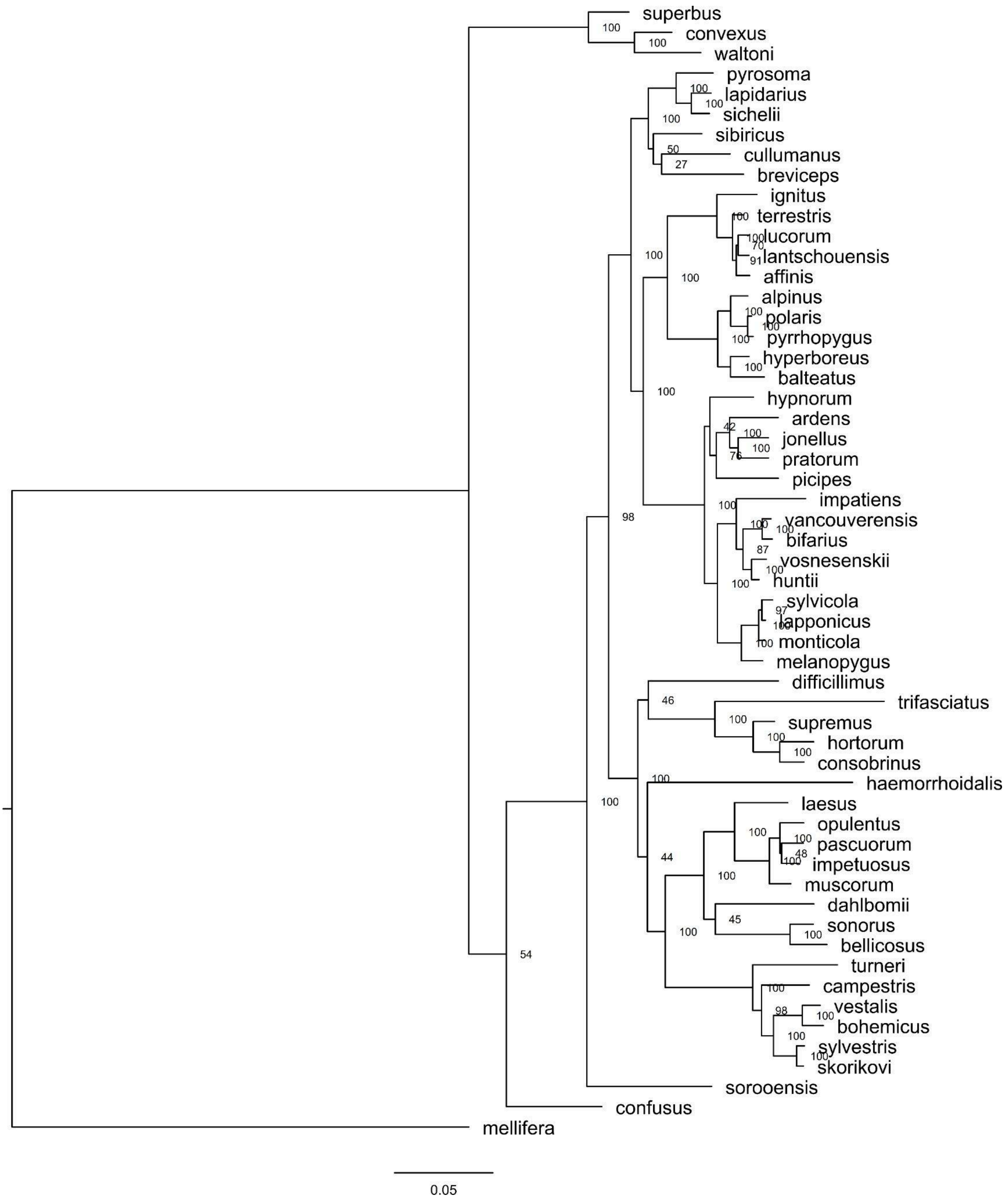

Supplementary figure 2. Unconstrained Maximum Likelihood phylogeny built using the concatenated amino acid matrix of N-mt genes. Scale bar indicates estimated substitutions per site. Only bootstrap values above 70 are shown.

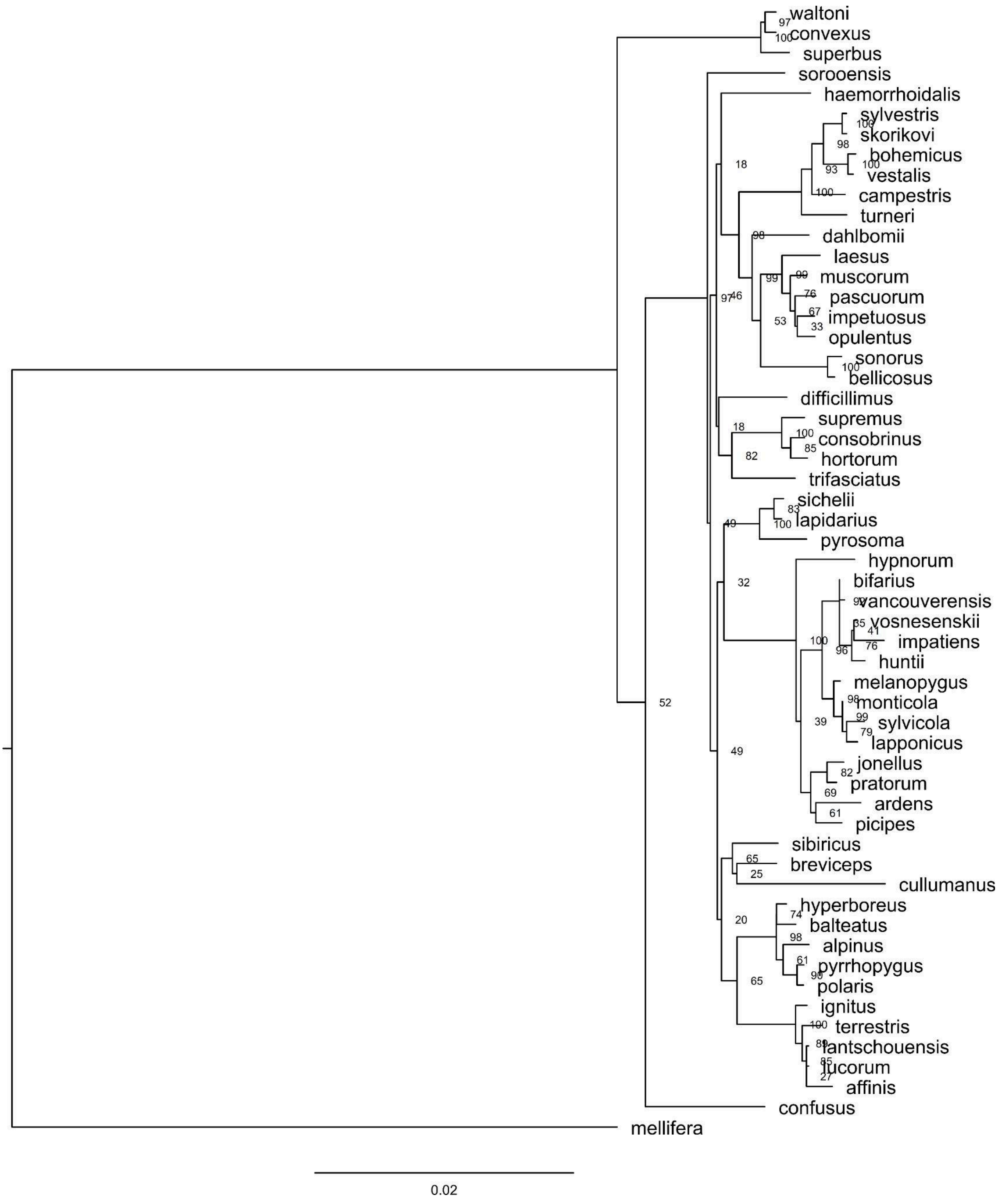

Supplementary figure 3. Unconstrained Maximum Likelihood phylogeny built using the concatenated amino acid matrix of glycolysis genes. Scale bar indicates estimated substitutions per site. Only bootstrap values above 70 are shown.

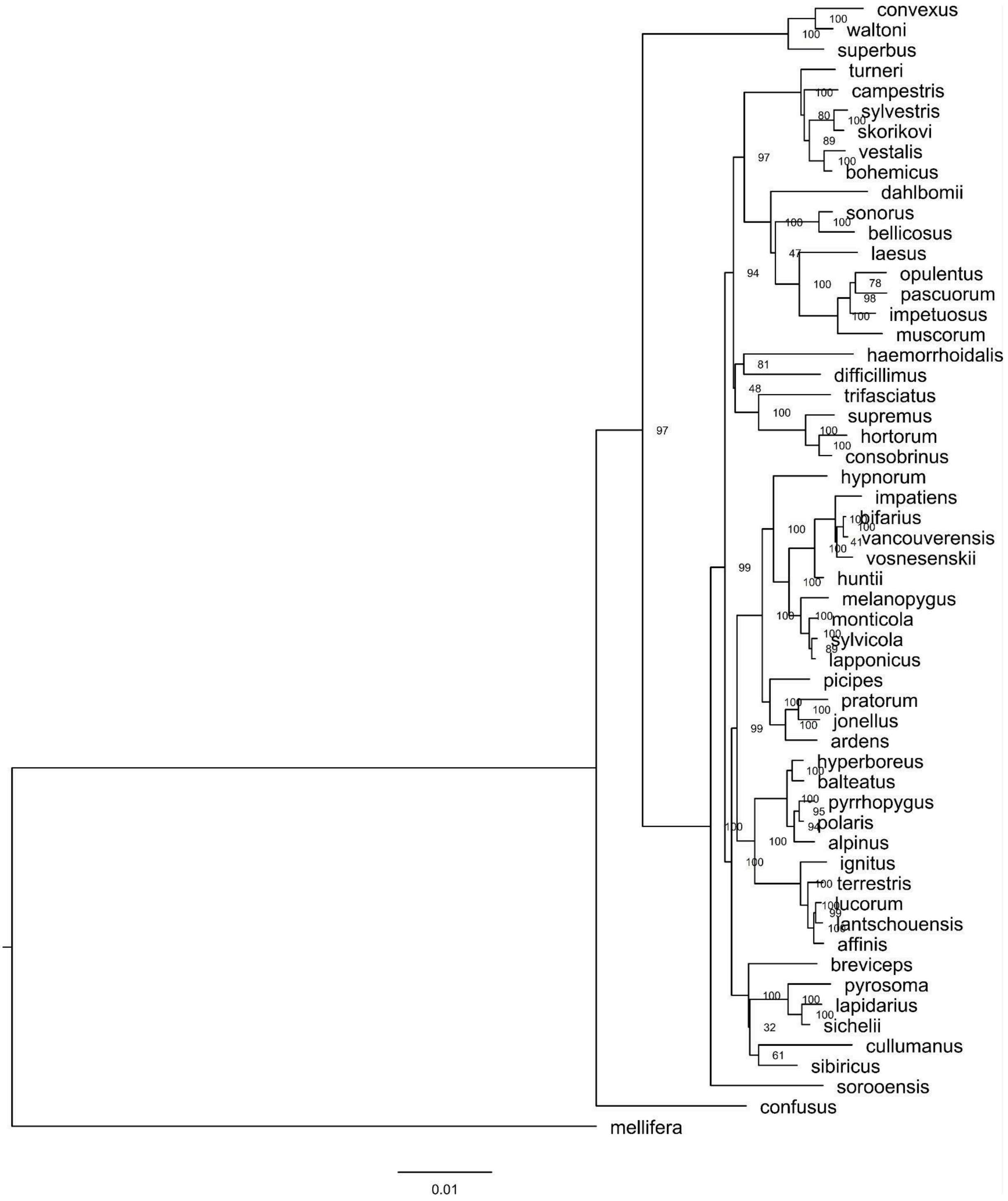

Supplementary figure 4. Unconstrained Maximum Likelihood phylogeny built using the concatenated amino acid matrix of random nuclear orthologs. Scale bar indicates estimated substitutions per site. Only bootstrap values above 70 are shown.
